# Cyclo-oxygenase inhibition reduces tumour growth and metastasis in an orthotopic model of breast cancer

**DOI:** 10.1038/sj.bjc.6600462

**Published:** 2002-07-02

**Authors:** E M Connolly, J H Harmey, T O'Grady, D Foley, G Roche-Nagle, E Kay, D J Bouchier-Hayes

**Affiliations:** Department of Surgery, Royal College of Surgeons in Ireland, Education and Research Centre, Beaumont Hospital, Dublin 9, Ireland; Department of Pathology, Royal College of Surgeons in Ireland, Education and Research Centre, Beaumont Hospital, Dublin 9, Ireland

**Keywords:** cyclo-oxygenase inhibitors, metastasis, breast cancer, angiogenesis, apoptosis

## Abstract

The effect of selective and non-selective cyclo-oxygenase inhibition on tumour growth and metastasis in an orthotopic model of breast cancer was investigated. 4T1 mammary adenocarcinoma cells were injected into the mammary fat pad of female BALB/c mice. When tumours reached a mean tumour diameter of 8.4±0.4 mm, mice were randomised into three groups (*n*=6 per group) and received daily intraperitoneal injections of the selective cyclo-oxygenase-2 inhibitor, SC-236, the non selective cyclo-oxygenase inhibitor, Indomethacin, or drug vehicle. Tumour diameter was recorded on alternate days. From 8 days after initiation of treatment, tumour diameter in animals treated with either SC-236 or indomethacin was significantly reduced relative to controls. Both primary tumour weight and the number of lung metastases were significantly reduced in the SC-236 and indomethacin treated mice. Microvessel density was reduced and tumor cell apoptosis increased in the primary tumour of mice treated with either the selective or non-selective cyclo-oxygenase inhibitor. *In vitro*, cyclo-oxygenase inhibition decreased vascular endothelial growth factor production and increased apoptosis of tumour cells. Our results suggest that cyclo-oxygenase inhibitors will be of value in the treatment of both primary and metastatic breast cancer.

*British Journal of Cancer* (2002) **87**, 231–237. doi:10.1038/sj.bjc.6600462 www.bjcancer.com

© 2002 Cancer Research UK

## 

Breast cancer is the commonest malignancy in women. Despite the introduction of breast screening and multimodal treatment with surgery, chemotherapy and radiotherapy over 14 000 women die every year in Britain from breast cancer (McPherson *et al*, 2000).

Angiogenesis, the formation of new capillaries from pre-existing blood vessels, is essential for the growth and metastasis of solid tumours (Folkman, 1990; Fidler and Ellis, 1994). High levels of angiogenesis within the primary tumour are associated with increased metastasis, as the new vessels allow tumour cells access to the circulation facilitating tumour dissemination (Anan *et al*, 1996). Angiogenesis is of particular importance in breast cancer where the switch to the angiogenic phenotype occurs at the pre-invasive ductal carcinoma *in situ* phase (Zolota *et al*, 1999). Microvessel density within the primary tumour, a measure of the degree of angiogenesis, is an independent predictor of metastatic disease in breast cancer (De Jong *et al*, 2000). Vascular endothelial growth factor (VEGF) is a potent pro-angiogenic cytokine (Peters *et al*, 1993) and increases vascular permeability, resulting in leaky blood vessels, which facilitates metastasis (Senger *et al*, 1983). We have recently demonstrated that VEGF also protects at least some breast cancer cells from apoptosis (Pidgeon *et al*, 2001). VEGF is produced by both host inflammatory cells and tumour cells (Freeman *et al*, 1995; Harmey *et al*, 1998). The level of tumour VEGF is an independent prognostic factor for survival in breast cancer (Linderholm *et al*, 1998).

In addition to new vessel formation, net tumour growth depends on the balance between tumour cell proliferation and apoptosis (Holmgren *et al*, 1995). Apoptosis, or programmed cell death, is characterised by single-cell death in the midst of living cells. The relative level of apoptosis and proliferation determines tumour growth, and any alteration in either process is a key element for determining tumour size (Fisher, 1994).

Prostaglandins are implicated in the development and growth of malignant tumours (Taketo, 1998). Cyclo-oxygenase (COX), the key regulatory enzyme for prostaglandin synthesis, is transcribed from two distinct genes. COX-1 is expressed constitutively in most tissues whereas COX-2 is rapidly induced at sites of inflammation and at sites of proliferation, for example within tumours (Bjorkman, 1998; Hawkey, 1999; Soslow *et al*, 2000). Non-steroidal anti-inflammatory drugs (NSAIDs) inhibit cyclooxygenase enzymes and consequently can inhibit, or abolish the effects of prostaglandins (Bjorkman, 1998; Hawkey, 1999). Increasing evidence shows that NSAIDs can inhibit tumour growth in experimental animals and in humans (Taketo, 1998; Milas, 1999). Commonly used NSAIDS, such as indomethacin, inhibit both COX-1 and COX-2 but treatment with such agents may be limited by toxicity to normal tissues, particularly ulceration and bleeding in the gastrointestinal tract as a consequence of COX-1 inhibition (Milas *et al*, 1999 and refs therein). Recently developed selective COX-2 inhibitors, for example celecoxib, exert potent anti-inflammatory activity but cause fewer unwanted side effects (Bjorkman, 1998; Hawkey, 1999). Selective COX-2 inhibitors have been shown to prevent carcinogenesis in experimental models of colon cancer (Reddy *et al*, 2000) and chemically-induced breast cancer (Harris *et al*, 2000). The possible mechanism of anti-tumour effects of COX inhibitors may be due to inhibition of angiogenesis (Tsujii *et al*, 1998) or induction of apoptosis (Li *et al*, 2001).

In this study the anti-tumour effects of a selective COX-2 inhibitor, SC-236, and the non-selective inhibitor, indomethacin were investigated in an orthotopic model of established breast cancer (Pulaski and Ostrand-Rosenberg, 1998). The direct effect of COX inhibition on tumour cell apoptosis and VEGF production *in vitro* was evaluated.

## MATERIALS AND METHODS

### Animals

Female 10- to 12-week old BALB/c mice (Charles River Institute, Margate, Kent, UK) were used. The animals were acclimatised for 1 week and caged in groups of five or less in an air conditioned room at ambient temperature of 21–22°C and 50% humidity under a 12-h light-dark cycle (lights at 08.00). Animals were housed in a licensed biomedical facility (RCSI Department of Surgery, Beaumont Hospital) and all procedures were carried out under animal license guidelines of the Department of Health, Ireland and in accordance with the UK Co-ordinating Committee on Cancer Research (UKCCR) Guidelines for the Welfare of Animals in Experimental Neoplasia. Animals had *ad libitum* access to animal chow (WM Connolly & Sons Ltd, Kilkenny, Ireland) and water.

### Tumour cells and culture conditions

4T1 tumour cells, a spontaneously metastasising mammary adenocarcinoma cell line were a generous gift from Dr Fred Miller, Duke University. Cells were maintained as monolayer cultures in Dulbecco's Modified Eagle Medium supplemented with 10% foetal bovine serum, sodium pyruvate, non-essential amino acids, L-glutamine and vitamins (Life Technologies, Inc., GIBCO–BRL, Paisley, UK) in an atmosphere of 5% CO_2_ in air at 37°C. Tumour cells were harvested from subconfluent cultures with 0.25% Trypsin-0.02% EDTA. Trypsin was neutralised with medium containing 10% FBS, washed three times in phosphate buffered saline (PBS) and resuspended in PBS at 5×10^5^ ml^−1^ for injection. Only single cell suspensions of greater than 90% viability as determined by Trypan blue exclusion were used for injections.

### Experimental design

Five×10^4^ (100 μl) 4T1 cells were injected into the mammary fat pad adjacent to the left forefoot after anaesthesia was induced and maintained with inhalational halothane. Primary tumours were measured on alternate days following injection of tumour cells using Vernier calipers. Tumour diameter (TD) was calculated as the square root of the product of two perpendicular diameters (Pulaski and Ostrand-Rosenberg, 1998). When mean TD was 8.4±0.4 mm (day 12 post injection of tumour cells), at which time micrometastases are already present in the lungs, mice were randomised into one of three groups (*n*=6 per group) to receive daily intraperitoneal injections of 200 μl vehicle (1% v v^−1^ dimethylsulphoxide [DMSO]), the selective COX-2 inhibitor, SC-236 (kind gift from Dr P Isakson, Monsanto, St Louis, MI, USA), (6 mg kg^−1^ in 1% DMSO) or the non-selective COX inhibitor, indomethacin (3 mg kg^−1^ in 1% DMSO). These doses were selected based on previously published studies (Milas *et al*, 1999), pilot studies and known toxicity of indomethacin. Tumour diameters were measured on alternate days. Thirteen days after initiation of drug treatment when the mean TD in the control group was 17 mm, all animals were euthanased. Primary tumours were excised, fixed in 10% formalin and processed for histology. Lungs were excised, fixed in Bouins solution and the number of lung lesions determined with the aid of a dissecting microscope (Yano *et al*, 2000).

At the time of sacrifice animals were anaesthesised with halothane and exsanguinated via closed cardiac puncture. Blood was allowed to clot for 2 h at room temperature and centrifuged for 20 min at 1100 **g**. Serum was removed, filtered through a 0.22 μm filter and stored at –80°C. VEGF was measured by enzyme-linked immunosorbent assay (ELISA) according to manufacturer's instructions (R&D Systems, Oxford, UK).

### Quantification of microvessel density

Paraffin embedded tissue sections (4 μm) were deparaffinised in xylene, rehydrated in graded alcohol, and transferred to PBS. Sections were washed twice with PBS, and endogenous peroxidase activity was blocked by incubating slides with 3% hydrogen peroxide in methanol. Sections were washed in PBS and incubated in blocking solution (5% normal rabbit serum in PBS) for 30 min. Excess blocking solution was aspirated and sections were incubated for 90 min at room temperature with 1 : 100 dilution of monoclonal goat anti CD31 (PECAM1-M20) (Santa Cruz, Biotech, Santa Cruz, CA, USA). Sections were washed with PBS followed by blocking solution for 10 min. Sections were washed with PBS and incubated with secondary antibody according to the manufacturer's instructions (Vectastain anti-goat kit, Vector labs). Bound antibody complexes were visualised using 3,3′-diaminobenzidine (DAB). Sections were counterstained with haematoxylin, dehydrated through graded alcohols, cleared with xylene and mounted in DPX mounting media.

For each section, vessels were counted in five high power fields (200×magnification (×20 objective and ×10 ocular)) as described (Xu *et al*, 2000). Data is expressed as mean±s.e.m.

### Tumour apoptosis and proliferation

Apoptotic cells in formalin fixed, paraffin-embedded breast tumour sections (4 μm), were stained using the in situ cell death detection kit according to the manufacturer's instructions (Boehringer Mannheim, East Sussex, UK). Peroxidase activity was visualised by the precipitation of DAB and sections were lightly counterstained with haematoxylin. Apoptotic cells stained brown against a blue background. Only cells that stained brown and had the morphological appearance of an apoptotic cell were counted as apoptotic cells, necrotic cells were easily distinguished and were excluded. Apoptosis was expressed as the number of positively stained cells in five high power fields per section (400×magnification (×40 objective and ×10 ocular)).

Proliferation in primary tumours was assessed following staining for proliferation cell nuclear antigen (PCNA). Four μm paraffin embedded sections were dewaxed in xylene, rehydrated in graded alcohols and washed in Tris-buffered saline (TBS) (25 mM Tris-HCl, pH 7.6, 150 mM NaCl) before blocking endogenous peroxidase activity in 3% hydrogen peroxide in methanol. Antigen retrieval was carried out by microwave treatment of sections in 10 mM trisodium citrate (pH 6.0) for 20 min. Sections were washed in TBS before incubation with mouse monoclonal anti-PCNA antibody (DAKO, Denmark) diluted 1 : 800 in TBS at room temperature for 40 min. Sections were washed in TBS and incubated with biotinylated goat anti mouse/rabbit immunoglobulins according to the manufacturer's instructions (StreptABComplex/HRP Duet kit [DAKO Corporation, Denmark]). Antibody complexes were visualised with DAB. Sections were counterstained with haematoxylin, dehydrated through graded alcohols, cleared with xylene and mounted in DPX mounting media. The percentage proliferation was calculated by counting the number of PCNA positive and PCNA negative cells in three high power fields per section (400×magnification (×40 objective and ×10 ocular)).

### *In vitro* experiments

#### VEGF production

Five×10^3^ 4T1 cells were plated at 5×10^3^/well in 96 well plates. 16 h later, SC-236 or indomethacin (5 or 10 μM each) were added for 24 h. Culture supernatants were collected and VEGF measured by ELISA (R&D Systems, UK). Cells were washed twice with PBS and total protein measured using the Bicinchonic Acid method (Pierce, IL, USA). VEGF was expressed as pg VEGF μg^−1^ cell protein. Each experiment was carried out three times in triplicate.

#### Apoptosis

Five×10^4^ 4T1 cells were plated on plastic culture chamber slides (LabTek™Permanox Chamber slides, Nalge Nunc International). Sixteen hours later SC-236 or indomethacin (5 or 10 μM each) were added for 24 h. Cells were fixed and stained using in situ cell death detection kit (Boehringer Mannheim, East Sussex, UK). The percentage of apoptotic cells per high power field (400×magnification (×40 objective and ×10 ocular)) was recorded in each of three fields per sample. Each experiment was carried out three times in triplicate.

### Statistical analysis

Data are expressed as mean±standard error mean (s.e.m.). Differences between *in vivo* and *in vitro* treatment groups were determined by one way ANOVA with Tukey Kramer *post-hoc* test using Instat for Windows statistics package (Graphpad Software Inc). Data were taken as significant where *P*<0.05.

## RESULTS

### Effect of COX inhibition on primary tumour growth and metastasis

**Figure 1:**
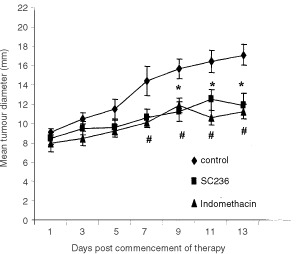
Tumour growth curve of mammary fat pad tumours. Twelve days after implantation of 4TI tumours, mice received daily injections of SC-236, indomethacin or vehicle for a further 13 days (*n*=6 per group). Tumour diameter was recorded on alternate days. Treatment with SC-236 or indomethacin significantly inhibited tumour growth relative to controls. Data expressed as mean±s.e.m. *P<0.05 COX-2 *vs* control, #*P*<0.05 indomethacin *vs* control.

4TI mammary adenocarcinoma cells spontaneously metastasise to the lungs from the mammary fat pad (Pulaski and Ostrand-Rosenberg, 1998). Following implantation of tumour cells into the mammary fat pad, tumour diameter (TD) was measured on alternate days. When mean TD was 8.4±0.4 mm, at which time micrometastases are already present in the lungs, treatment with 6 mg kg^−1^ SC-236 (15.6 μM) or 3 mg kg^−1^ indomethacin (8.3 μM) was initiated. These doses were based on pilot studies and previously published doses (Milas *et al*, 1999). Although 3 mg kg^−1^ indomethacin has previously been found to be toxic when administered by gavage (Masferrer *et al*, 2000), we did not observe any toxicity using intra-peritoneal delivery. From 8 days after treatment until the end of the experiment, there was a significant difference in primary tumour growth in the SC-236-treated and indomethacin-treated mice relative to untreated control mice. There was no significant difference in TD of mice treated with either the selective COX-2 inhibitor, SC-236 (6 mg kg^−1^), or the non-selective COX inhibitor, indomethacin (3 mg kg^−1^) (Figure 1

). Neither drug regime caused regression of the established tumours but rather inhibited further tumour growth. Thirteen days after treatment was initiated, there was a significant reduction (*P*<0.05) in primary tumour size in both SC-236 treated (11.8±1.3 mm) and indomethacin treated mice (11.2±0.7 mm) relative to control (17.0±1.1 mm) (Table 1

**Table 1:**
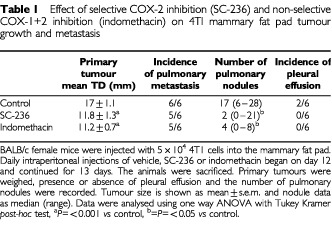
Effect of selective COX-2 inhibition (SC-236) and non-selective COX-1+2 inhibition (indomethacin) on 4TI mammary fat pad tumour growth and metastasis

). There was no significant difference in tumour size between SC-236 and indomethacin treated groups.

Both SC-236 and indomethacin treatment resulted in a significant reduction in the number of spontaneous lung metastases relative to untreated controls (Table 1). Pleural effusions were present in two of the control mice whereas none of the mice in the treatment groups had evidence of pleural effusions (Table 1). The effects of COX inhibition on primary tumour growth and metastasis were confirmed in a second experiment (*n*=5 per group, data not shown).

### Effect of COX inhibitors on proliferation and apoptosis within the primary tumour

**Figure 2:**
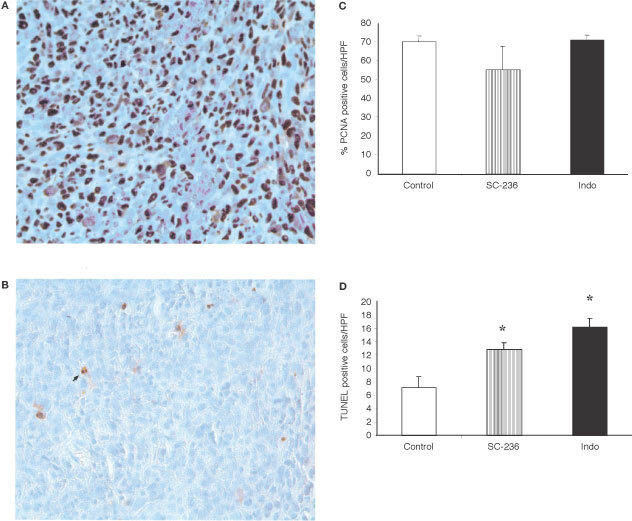
Apoptosis and proliferation in 4TI mammary fat pad tumours. (**A**) PCNA-positive (proliferating) cells within tumours (original magnification ×400). (**B**) TUNEL-positive (apoptotic) cells within tumours. Apoptotic cells stain brown after TUNEL staining (original magnification×400). (**C**) % PCNA positive cells in tumours from control mice and mice treated with SC-236 or indomethacin. Three high power fields per section (400×magnification (×40 objective and ×10 ocular)). Data represented as mean±s.e.m. *P*=n.s. (**D**) Number of TUNEL positive cells per h.p.f. Five high power fields per section (400×magnification (×40 objective and ×10 ocular)). SC-236 or indomethacin significantly increased apoptosis relative to tumours from control mice (one section from each of six mice per group). Data represented as mean±s.e.m. *P<0.05.

The relative levels of apoptosis and cell proliferation determine net tumour growth. As both the selective and non-selective COX inhibitors significantly reduced primary tumour growth we investigated the effects of COX inhibition on apoptosis and proliferation within the tumour. Proliferating cells were identified by PCNA staining and apoptosis by TUNEL staining. Representative sections stained for PCNA and apoptotic cells are shown in Figure 2A,B

, respectively. Neither treatment had any effect on level of proliferation within the tumour (Figure 2C). However, both SC-236 (12.75±0.98 apoptotic cells per h.p.f.) and indomethacin (16.1±1.3 apoptotic cells per h.p.f.) treatment results in a significant increase in the level of apoptosis relative to control tumours (7.12±1.6 apoptotic cells per h.p.f.) (Figure 2D) with no significant difference between the two treatment groups.

### COX inhibition reduces tumour angiogenesis

**Figure 3:**
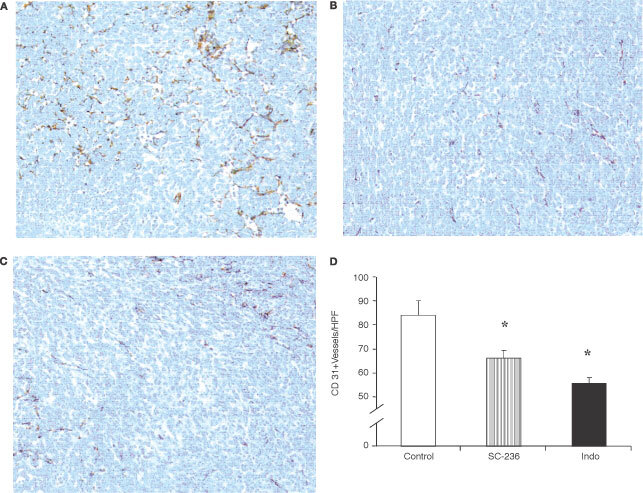
Angiogenesis in 4TI mammary fat pad tumours. Representative sections of tumours stained for CD31 from control mice (**A**), SC-236 treated (**B**), and indomethacin treated mice (**C**) are shown. Original magnification×200. (**D**) Microvessel density was assessed by light microscopy following CD31 staining (one section from each of six mice per group). For each section, vessels were counted in five high power fields (200×magnification (×20 objective and ×10 ocular)). Data is expressed as mean number of vessels per h.p.f.±s.e.m. *P<0.05 *vs* control.

To further investigate the mechanisms by which treatment with either a selective COX-2 inhibitor (SC-236) or a non-selective COX-1 and COX-2 inhibitor (indomethacin) reduced primary tumour growth and metastasis, we examined the effect of treatment on angiogenesis within the primary tumour. Vascularisation was identified by staining tumours with an antibody to CD31 and the number of vessels per high power field scored. Representative sections from control, SC-236 treated and indomethacin treated tumours are shown in Figure 3A–C

respectively. Both SC-236 (65.8±3.5 vessels per h.p.f.) and indomethacin (55.6±2.2 vessels per h.p.f.) treatment significantly reduced angiogenesis in the primary tumour relative to controls (84±6 vessels per h.p.f.) (Figure 3D). There was no significant difference in microvessel density within SC-236 or indomethacin treated tumours.

**Figure 4:**
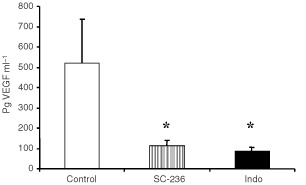
Serum VEGF levels. Blood was collected by cardiac puncture and serum VEGF measured by ELISA (*n*=6 per group). Data is expressed as mean pg VEGF ml^−1^ serum±s.e.m. Treatment with SC-236 or indomethacin significantly reduced VEGF relative to controls. *P<0.05.

Serum VEGF was measured by ELISA (Figure 4

). Treatment with either SC-236 (114±23.6 pg ml^−1^) or indomethacin (87.2±18.6 pg ml^−1^) significantly reduced circulating VEGF relative to controls (516.4±215 pg ml^−1^).

### Effect of COX inhibition on 4T1 cell apoptosis and VEGF production *in vitro*

**Figure 5:**
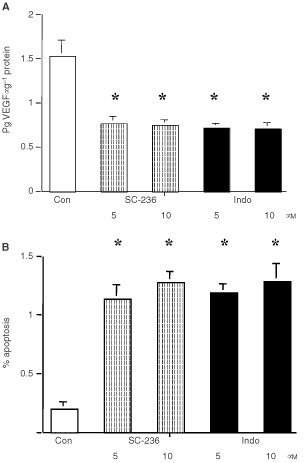
*In vitro* experiments. (**A**) VEGF production by 4T1 cells. SC-236 or Indomethacin at 5 or 10 μM significantly reduced VEGF production (pg VEGF μg^−1^ total protein) relative to controls (*P<0.001 in all cases). There was no significant difference between SC-236 or indomethacin treatment at either dose. Data represent mean±s.e.m. of three experiments carried out in triplicate. (**B**) 4T1 apoptosis. SC-236 or Indomethacin at 5 or 10 μM significantly increased the percentage of apoptotic cells relative to controls (*P<0.001 in all cases). There was no significant difference between SC-236 or indomethacin treatment at either dose. Data represent mean±s.e.m. of three experiments carried out in triplicate.

*In vivo*, treatment with either indomethacin (8.3 μM) or SC-236 (15.6 μM) resulted in increased apoptosis within primary tumour and decreased circulating VEGF. We examined the effects of these compounds on apoptosis and VEGF production by 4T1 cells *in vitro*. Treatment with SC-236 (5 μM: 0.77±0.066 pg μg^−1^, 10 μM: 0.74±0.063 pg μg^−1^)or indomethacin (5 μM: 0.71± 0.058 pg μg^−1^, 10 μM: 0.71±0.066 pg μg^−1^), significantly decreased VEGF production by 4T1 tumour cells relative to control cells (1.5±0.18 pg μg^−1^) (Figure 5A

). There was no significant difference between SC-236 and indomethacin at either of the doses studied. Furthermore, when we examined tumour cell apoptosis, we found that either SC-236 (5 μM: 1.14±0.119%, 10 μM: 1.28±0.090%), or indomethacin (5 μM: 1.19±0.072%, 10 μM: 1.29±0.149%), treatment significantly increased apoptosis at both 5 and 10 μM relative to control cells (0.2±0.059% apoptosis) and again, there was no difference between the two inhibitors at either of the doses studied (Figure 5B).

## DISCUSSION

Using an orthotopic murine model of breast cancer, cyclo-oxygenase inhibitors reduced primary tumour growth and spontaneous metastasis, an effect associated with increased apoptosis and reduced angiogenesis in the primary tumour.

Although NSAIDs have been shown to reduce the risk of breast cancer (Sharpe *et al*, 2000), it is not known how they reduce this risk nor is it known what effect they have on established breast cancer. Both selective COX-2 (celecoxib) and non selective COX inhibition (ibuprofen) reduced the incidence and subsequent growth of primary breast tumours induced by 7,12-dimethylbenz(a)anthracene (DMBA) in female Sprague Dawley rats (Alshafie *et al*, 2000; Harris *et al*, 2000). The selective COX-2 inhibitor, celecoxib, inhibited the growth and metastasis of ectopically implanted Lewis lung and HT29 colon tumours (Masferrer *et al*, 2000). However, it is well established that faithful reproduction of the tumour microenvironment occurs only in orthotopic tumour models (Killion *et al*, 1999; Hoffman, 1999). In this experiment we used an orthotopic murine model of breast cancer where 4TI mammary carcinoma cells were injected directly into the mammary fat pad of female BALB/c mice. 4TI cells originated from a spontaneously occurring BALB/c mouse breast tumour and share many characteristics with human mammary cancers, making it an excellent model of breast cancer (Pulaski and Ostrand-Rosenberg, 1998). This model also has the advantage of forming spontaneous metastases and pleural effusions similar to the development of malignant pleural effusions in advanced breast cancer patients. In this model, either the selective COX-2 inhibitor, SC-236, or the non selective COX inhibitor, indomethacin, inhibited the growth of established tumours. The number of spontaneous lung metastases was also significantly reduced following either treatment. This reduction in metastasis could be a consequence of reduced primary tumour size in treated mice rather than an inhibition of metastasis *per se*. We are currently examining the effect of COX inhibitors on the growth of metastases following excision of a primary tumour and in experimental metastasis models to clarify this issue. It will also be necessary to examine the efficacy of COX inhibitors in human orthotopic models.

NSAIDS can induce cells to undergo apoptosis *in vitro* (Lu *et al*, 1995). Selective COX-2 inhibition has been shown to induce apoptosis through a cytochrome C-dependent pathway in oesophageal cancer cells (Li *et al*, 2001). Arachidonic acid, the substrate for COX, stimulates apoptosis, thus enhanced COX-2 expression could inhibit apoptosis by decreasing arachidonic acid (Cao *et al*, 2000). We found that *in vivo*, COX inhibition increased apoptosis with no change in proliferation in the primary tumours relative to control. Furthermore, COX inhibition *in vitro* directly increased tumour cell apoptosis.

Microvessel density within the primary tumour has been shown to be an independent predictor of metastatic disease in breast cancer patients (De Jong *et al*, 2000). COX-2 inhibitors have been shown to reduce angiogenesis *in vitro* (Tsujii *et al*, 1998). Williams *et al* (2000) found reduced angiogenesis in Lewis lung carcinomas grown in COX-2 knockout (COX-2^−/−^) mice when compared to tumours grown in wild type mice. In our study, inhibition of primary tumour growth and metastasis in mice treated with COX inhibitors was associated with a significant reduction in microvessel density in the primary tumour, suggesting that these drugs exert their anti-tumour effect, at least in part, by reducing angiogenesis in the primary tumour. As the degree of tumour angiogenesis is predictive of metastatic disease (De Jong *et al*, 2000), the reduction in primary tumour MVD may account for the reduced number of pulmonary metastases following COX inhibition. We also found reduced serum levels of VEGF in the COX inhibited groups relative to control. However, we previously demonstrated that serum VEGF correlates with metastatic burden in an experimental metastasis model (Pidgeon *et al*, 1999). Both the reduction in VEGF and angiogenesis could simply be a reflection of reduced primary tumour size in treated mice, although *in vitro* COX inhibition directly reduced VEGF production by the 4T1 tumour cells used in this study.

These results suggest that COX inhibitors target two of the central balances governing tumour growth and metastasis, namely angiogenesis and apoptosis. In this study for all parameters studied, the selective COX-2 inhibitor, SC-236, was as effective as the non-selective inhibitor, indomethacin, at the doses examined, suggesting that the anti-tumour effects observed herein with indomethacin, are primarily due to the inhibition of COX-2. This suggests that selective COX-2 inhibition may be as effective as the non-selective NSAIDs with the distinct advantage of a low toxicity profile (Hawkey, 1999, Masferrer *et al*, 2000). The anti-angiogenic and pro-apoptotic properties of COX inhibitors, suggest that these drugs may increase the anti-tumour effects of conventional chemotherapy and radiotherapy used in the treatment of breast cancer. Indeed, SC-236 has been shown to increase radiosensitivity in a murine sarcoma model (Milas *et al*, 1999).
